# Commentary: Regulatory Innate Lymphoid Cells Control Innate Intestinal Inflammation

**DOI:** 10.3389/fimmu.2018.01522

**Published:** 2018-07-02

**Authors:** Boning Zeng, Shengnan Shi, Jing Liu, Feiyue Xing

**Affiliations:** ^1^Department of Immunobiology, Institute of Tissue Transplantation and Immunology, Jinan University, Guangzhou, China; ^2^Key Laboratory of Functional Protein Research of Guangdong, Higher Education Institutes, Jinan University, Guangzhou, China; ^3^School of Stomatology, Jinan University, Guangzhou, China

**Keywords:** regulatory innate lymphoid cells, innate lymphoid cell, development, regulation, inflammatory bowel disease

Over the past decade, innate lymphoid cells (ILCs) have been identified as a subpopulation of innate immune cells. These cells exist mostly in intestinal mucosal tissue and express no lymphoid differentiation lineage negative (Lin^−^) markers and antigen-specific receptors that make them distinct from T cells or B cells (1). They were originally classified as ILC1, ILC2, and ILC3 based on their phenotypic features and functions (2). ILC1 cells secrete interferon-γ (IFN-γ) after interleukin-12 (IL-12), IL-15, and IL-18 stimulation and express the transcription factor T-bet (3). ILC2 cells mainly produce the type 2 T helper cells like cytokines IL-5, IL-9, and IL-13 in response to the stimulation of IL-25 and IL-33 in the presence of the transcriptional factor GATA3 (4). ILC3 cells largely generate IFN-γ, IL-17, and IL-22 under the stimulation of IL-1 and IL-23 in the presence of the transcription factors RORγt and aryl hydrocarbon receptor (AhR) (5). Hence, the ILCs participate in the formation of mucosal immunity, development of lymphoid cells, rehabilitation of tissue injury, and the protection of epithelial barriers.

However, in a recent study published in *Cell* (6), Lin^−^CD45^+^CD127^+^IL-10^+^ cells were, for the first time, recognized to represent a novel subset of the IL-10-producing ILCs in mice, called regulatory ILCs (ILCreg). These are mostly present in the gut tissue and differentiate and mature in response to pathogen stimulation. In comparison with other ILCs or regulatory T cells (Treg), the ILCreg possess an exclusive gene identity. They express neither CD4 nor FoxP3, the signature marker of Treg cells (6), but are morphologically similar to lymphoid cells with a high nuclear to cytoplasmic ratio. In addition, the authors reported that the ILCreg expressed various phenotypic ILC markers, including CD25 (IL-2Rα), Sca-1, and CD90 (Thy1), but not ILC1 (NK1.1 and NKp46), ILC2 (ST2 and KLRG1), or ILC3 markers (NKp46, CD4, and RORγt) in inflamed intestinal tissue. The ILCreg inhibited the activation of ILC1 and ILC3 *via* secretion of IL-10 but did not affect ILC2 with undetectable *Il10rb* expression, showing that it is a novel ILC subpopulation discriminated from other ILC subsets.

Interestingly, Seehus et al. (7) described a distinct subpopulation of IL-10-producing lung ILC2 cells, termed ILC2_10_, that can suppress pathogenic inflammatory immune responses. Unlike most lung ILC2 with the Thy1.1-negative phenotype, the ILC2_10_ are Thy1.1-positive, generate IL10 to exert modulating roles under the stimulation of IL-33 and downregulate proinflammatory genes, such as Tnf, Lta, Il2, and Ccl1. The ILC2_10_ produce higher levels of transcription factors Id3, Foxf1, Atf3, and Klf2 than ILC2. IL-33 and IL7 instruct ILC2 to produce IL-13, but have no effect on the ILC2_10_ *in vitro*. Analogous to the ILCreg, the ILC2_10_ have no expression of Foxp3, and IL-2 signaling promotes IL-10 production from ILC2_10_. However, they also express the anti-inflammatory gene Retnla, suggesting that the ILC2_10_ may possess anti-inflammatory properties. Interestingly, the ILC2_10_ exhibit memory-like properties *in vivo*. The ILC2_10_ are in a resting status after *in vivo* stimulation is discontinued, but are swiftly reactivated and expanded once again when re-stimulated by a minimal dose of IL-33, similar to memory T cells (7). In addition, a third regulatory subset, CD56^+^CD3^−^ ILCs were recently described to have regulatory functions in human and mice. They simultaneously express NK cell- and ILC-associated molecules, including CD56, CD94, KLRK1 (NKG2D), killer cell Ig-like receptors (KIRs), NCR3 (NKP30), and NCR1 (NKP46). Consistent with the ILCreg, the CD56^+^CD3^−^ ILCs express no transcription factor Foxp3, but produce high levels of CD94, various KIRS, EOMES, TBX21, GATA3, RORA, and AhR, suggesting the existence of transcriptional factor profiles overlapping with NK cells, ILC2, and ILC3 (8).

A restricted common helper-like innate lymphoid progenitor (CHILP) express the transcriptional factor PLZF and differentiate into the downstream common ILC precursor (ILCP) that give rise to formation of ILC1, ILC2, and ILC3 subsets (9–11), in which ILC2 are a major ILC population in the lungs to play an important role in protecting airway and lung tissue from virus infection (12). Wang et al. (6) demonstrated that the ILCreg originate from CHILP and not ILCPs within the gut tissue (Figure 1). Dissimilarly, the ILC2_10_ are derived from ILCP, but CD56^+^CD3^−^ ILCs may originate from NK cells (8). Of note, although all these three subsets are considered to display regulatory roles, they appear to be distinct roles as the ILCreg can relieve intestinal inflammation, ILC2_10_ regulate chronic inflammatory conditions, including allergies, whereas CD56^+^CD3^−^ ILCs inhibit tumor-infiltrating lymphocytes. Neither the ILCreg, ILC2_10_, nor the CD56^+^CD3^−^ ILCs express the transcription factor Foxp3. However, both the ILCreg and the ILC2_10_ express the transcription factor ID3, which contains a HLH dimerization domain but lacks the basic DNA binding region, to inhibit inflammation (13). The CD56^+^CD3^−^ ILCs and the ILC2_10_ express the transcription factor GATA3 and RORα. Unlike ILCreg and ILC2_10_, the CD56^+^CD3^−^ ILCs produce IL-22 but not IL10, with expressions of ILC3 transcription factor AhR and NK cell transcription factor EOMES. It is likely that the different origins and distributions of these three subsets are responsible for their distinct constitutive expressions of transcription factors and cytokines, and hence their functional diversity.

**Figure 1:**
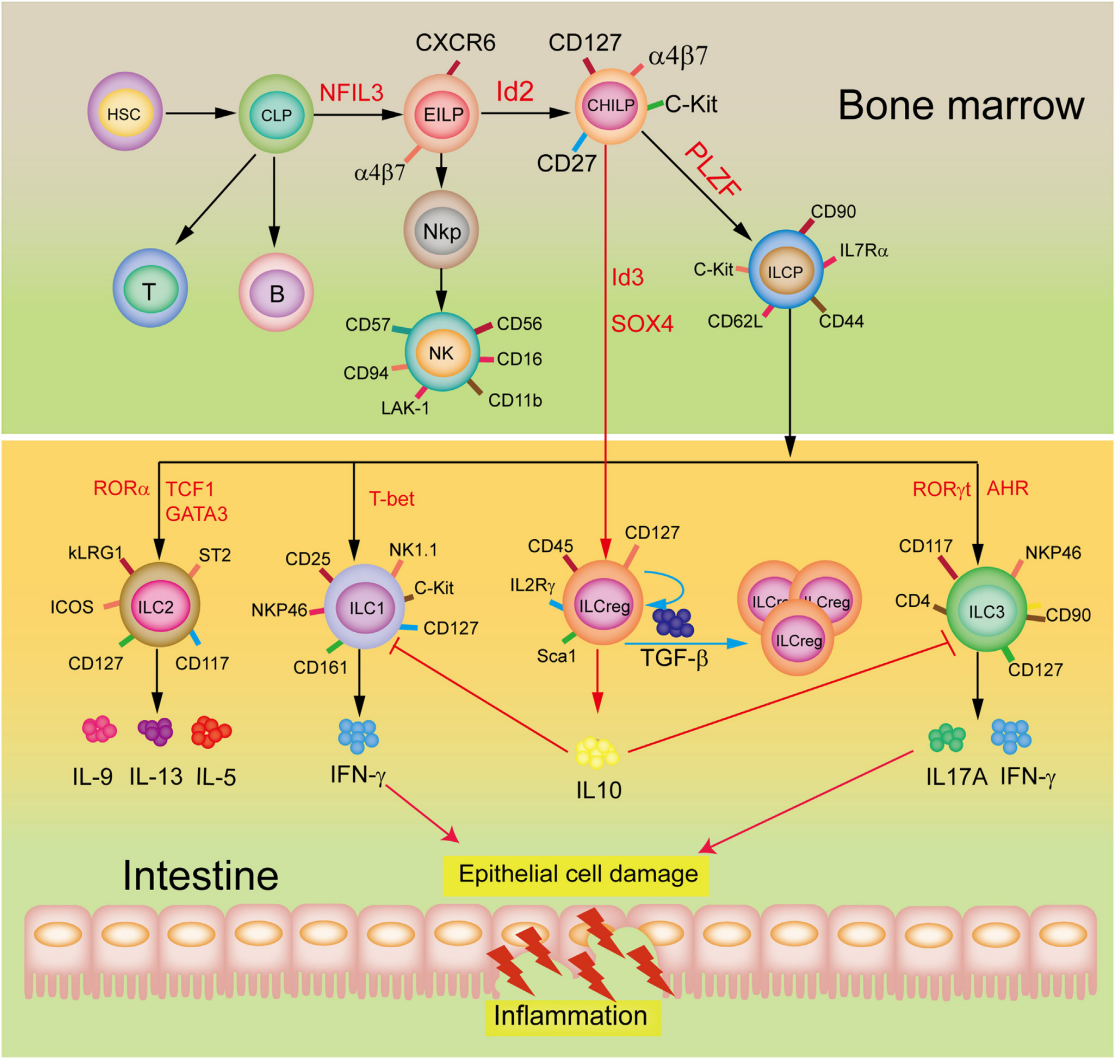
Development and potential functions of regulatory innate lymphoid cells (ILCreg). The ILCreg originate from CHILP not ILCPs and depend on the transcriptional factors Id3 and SOX4. The ILCreg can inhibit the activation of ILC1 and ILC3 to relieve innate intestinal inflammation *via* IL-10 secretion, and promote self-expansion by secreting TGF-β during inflammation. Abbreviations: CLP, common lymphoid progenitor; CHILP, common helper-like innate lymphoid precursor; EILP, early innate lymphoid precursor; PLZF, promyelocytic leukemia zinc finger; NKP, NK cell progenitor; AhR, aryl hydrocarbon receptor; NFIL3, nuclear factor interleukin 3; GATA3, GATA-binding protein 3; ID2, inhibitor of DNA binding 2; ILCP, ILC precursor; ROR, retinoic acid receptor-related orphan receptor; IFN-γ, interferon-γ; ID3, inhibitor of DNA binding 3; SOX4, sex-determining region Y-box 4; TCF1, T cell factor-1; T-bet, T-box expressed in T cells; TGF-β, transforming growth factor-β.

These new findings not only advances our understanding of intestinal mucosal microenvironment balance but also diseases such as the inflammatory bowel disease, and may contribute significantly toward developing therapeutic applications. Emergence of ILCreg as a novel ILC subset also raises new interesting questions. How do Id3 and SOX4 affect the ILCreg development and fate? Since Treg can generate IL-10 in responses to IL-2 and TGF-β stimulation, is it possible that the ILCreg may be activated by both IL-2 and TGF-β signaling. A mechanistic process by which the ILCreg secrete IL-10 also needs further exploration. Additionally, the populations of ILC1 and ILC3 are obviously increased in the inflamed intestinal mucosa (14). The ILCreg suppress the activation of ILC1 and ILC3 by secreting a high level of IL-10 but why does it not impact ILC2 during intestinal inflammation? An exploration of these will provide a novel insight into the fundamental roles and mechanisms of maintenance of gut innate immune homeostasis and defense against inflamed intestinal mucosa injury.

## Author Contributions

FX conceived of this commentary. FX, BZ, and SS drafted it. JL and FX finalized the manuscript.

## Conflict of Interest Statement

The authors declare that the research was conducted in the absence of any commercial or financial relationships that could be construed as a potential conflict of interest.

## References

[1] Morita H Moro K Koyasu S. Innate lymphoid cells in allergic and nonallergic inflammation. J Allergy Clin Immunol (2016) 138(5):1253–64. 10.1016/j.jaci.2016.09.011 27817797

[2] Ebihara T Song C Ryu SH Plougastel-Douglas B Yang L Levanon D Runx3 specifies lineage commitment of innate lymphoid cells. Nat Immunol (2015) 16(11):1124–33. 10.1038/ni.3272 26414766 PMC4618046

[3] Bernink JH Krabbendam L Germar K de Jong E Gronke K Kofoed-Nielsen M Interleukin-12 and -23 control plasticity of CD127(+) group 1 and group 3 innate lymphoid cells in the intestinal lamina propria. Immunity (2015) 43(1):146–60. 10.1016/j.immuni.2015.06.019 26187413

[4] Brestoff JR Kim BS Saenz SA Stine RR Monticelli LA Sonnenberg GF Group 2 innate lymphoid cells promote beiging of white adipose tissue and limit obesity. Nature (2015) 519(7542):242–6. 10.1038/nature14115 25533952 PMC4447235

[5] Magri G Cerutti A. Role of group 3 innate lymphoid cells in antibody production. Curr Opin Immunol (2015) 33:36–42. 10.1016/j.coi.2015.01.008 25621842 PMC4488900

[6] Wang S Xia P Chen Y Qu Y Xiong Z Ye B Regulatory innate lymphoid cells control innate intestinal inflammation. Cell (2017) 171(1):201–16.e18. 10.1016/j.cell.2017.07.027 28844693

[7] Seehus CR Kadavallore A Torre B Yeckes AR Wang Y Tang J Alternative activation generates IL-10 producing type 2 innate lymphoid cells. Nat Commun (2017) 8(1):1900. 10.1038/s41467-017-02023-z 29196657 PMC5711851

[8] Crome SQ Nguyen LT Lopez-Verges S Yang SY Martin B Yam JY A distinct innate lymphoid cell population regulates tumor-associated T cells. Nat Med (2017) 23(3):368–75. 10.1038/nm.4278 28165478 PMC5497996

[9] Diefenbach A Colonna M Koyasu S. Development, differentiation, and diversity of innate lymphoid cells. Immunity (2014) 41(3):354–65. 10.1016/j.immuni.2014.09.005 25238093 PMC4171710

[10] Liu M Zhang C. The role of innate lymphoid cells in immune-mediated liver diseases. Front Immunol (2017) 8:695. 10.3389/fimmu.2017.00695 28659927 PMC5468686

[11] Yang Q Bhandoola A. The development of adult innate lymphoid cells. Curr Opin Immunol (2016) 39:114–20. 10.1016/j.coi.2016.01.006 26871595 PMC4801723

[12] Lai D Tang J Chen L Fan EK Scott MJ Li Y Group 2 innate lymphoid cells protect lung endothelial cells from pyroptosis in sepsis. Cell Death Dis (2018) 9(3):369. 10.1038/s41419-018-0412-5 29511181 PMC5840374

[13] Miyazaki M Miyazaki K Chen S Itoi M Miller M Lu LF Id2 and Id3 maintain the regulatory T cell pool to suppress inflammatory disease. Nat Immunol (2014) 15(8):767–76. 10.1038/ni.2928 24973820 PMC4365819

[14] Fuchs A Vermi W Lee JS Lonardi S Gilfillan S Newberry RD Intraepithelial type 1 innate lymphoid cells are a unique subset of IL-12- and IL-15-responsive IFN-gamma-producing cells. Immunity (2013) 38(4):769–81. 10.1016/j.immuni.2013.02.010 23453631 PMC3634355

